# The Combination of AFP and “Up-To-Seven” Criteria May Be a Better Strategy for Liver Transplantation in Chinese Cirrhotic HCC Patients

**DOI:** 10.3389/fonc.2022.959151

**Published:** 2022-07-12

**Authors:** Da-li Zhang, Dan-ni Feng, Xi He, Xiao-feng Zhang, Li-xin Li, Zhi-jie Li, Xiao-feng Niu, Yun-long Zhuang, Zhen-wen Liu, Xu-dong Gao, Hong-bo Wang

**Affiliations:** Department of Hepatology, The Fifth Medical Center of Chinese People’s Liberation Army General Hospital, Beijing, China

**Keywords:** liver transplantation, hepatocellular carcinoma, Up-to-seven criteria, alpha-fetoprotein, beyond Milan criteria, long-term outcomes

## Abstract

**Background:**

Orthotopic liver transplantation (OLT) is a life-saving option for patients with hepatocellular carcinoma (HCC), but the expanded OLT criteria remain controversial.

**Objective:**

The study aimed to explore whether expanded OLT criteria can be applied to Chinese cirrhotic patients with HCC.

**Methods:**

This retrospective study analyzed risk factors for HCC recurrence and death and compared patients’ tumor characteristics and outcomes in groups of Milan, “Up-to-seven,” and Hangzhou criteria, and groups between met and unmet the combinative criteria of “Up-to-seven” and AFP of < 1000 ng/mL.

**Results:**

Among 153 patients who underwent OLT for HCC from January 2015 to February 2019 in 4 years of follow-up, 20 (13.1%) patients had HCC recurrence, and 11 (7.2%) had HCC-related death. Multivariate Cox regression analysis showed that preoperative alpha-fetoprotein (AFP) of > 1000 ng/mL (hazard ratio [HR]: 10.05, 95% confidence interval [CI]: 2.45–41.13, P = 0.001) was an independent risk factor for HCC recurrence and HCC-related death (HR: 6.63, 95%CI: 1.31–33.52, P = 0.022). Patients who did not meet Milan criteria but satisfied the “Up-to-seven” criteria had no differences in overall survival (OS) (P = 0.69) and disease-free survival (DFS) (P = 0.35) than patients who met the Milan criteria. The combination of “Up-to-seven” criteria and AFP of < 1000 ng/mL differed significantly (HR: 18.9; 95% CI: 4.0–89.2; P < 0.001). Patients with HCC who met the “Up-to-seven” criteria and AFP of < 1000 ng/mL (n = 121) had excellent survival with 4-year OS of 91.6% (P < 0.001) and DFS of 90.8% (P < 0.001), which is significantly better compared to the other group (n = 32) (OS of 67.5% and DFS of 46.5%) and patients who met the Milan criteria (n = 108, OS of 89.8%, DFS of 89.6%), allowing 28.9% (13/45) of patients who did not meet the Milan criteria to benefit from OLT.

**Conclusion:**

Chinese cirrhotic patients with HCC who met the combinative criteria of “Up-to-seven” and AFP of < 1000 ng/mL had better survival than those who met the Milan criteria, and these combinative criteria benefited more patients and may become a better option for OLT.

## Introduction

Hepatocellular carcinoma (HCC) is one of the most common causes of cancer-related death worldwide (1). Hepatic resection is the most widely applied treatment option, but it has a high incidence of tumor recurrence of 70% at 5 years and may cause complications of chronic liver disease (2). Orthotopic live transplantation (OLT) is a curative therapy for HCC, not only removing the tumor burden but also the native cirrhotic liver. Thus, OLT is the most effective treatment for HCC.

According to the Barcelona Clinic Liver Cancer (BCLC) treatment strategy, patients with > 1 nodules should consider OLT (3). Milan criteria proposed by Professor Mazzaferro are a metric for OLT and defined by a single tumor diameter of ≤ 5 cm, multiple tumor nodules of ≤ 3, each nodule of ≤ 3 cm, no vessel invasion, and no metastases (4). Milan criteria are restrictive: many patients who might obtain good survival after liver transplantation do not have the opportunity to be OLT candidates (5–7). Therefore, more expanded OLT criteria have been explored to benefit more patients with HCC. However, patients with expanded OLT criteria had lower overall survival (OS) and disease-free survival (DFS) than those with Milan criteria (8). A growing number of studies try to improve the OS and DFS by combining other markers or building new models, but there is no consensus (9–12).

“Up-to-seven” criteria are defined by the number of tumors and the sum of tumor diameter of ≤ 7 and were proposed by Professor Mazzaferro in 2009 (13), with a 5-year survival of 71.2%, being easy to calculate. Recently, “Up-to-seven” has been reported as an accurate tool to predict the prognosis of HCC patients undergoing hepatic resection, transcatheter arterial chemoembolization (TACE), and systemic therapies. However, these criteria do not include alpha-fetoprotein (AFP), the most important marker of HCC recurrence. High AFP is a well-known predictive factor of poor prognosis, and AFP levels of > 1000 ng/mL have been associated with a high risk of recurrence; also, its exclusion from OLT has been suggested (14–16). AFP of > 400 ng/mL is a risk for HCC recurrence in Hangzhou criteria (17); the AFP model and Metroticket 2.0 model are based on AFP level (18). Thus, AFP is vital for HCC recurrence. Whether the combination of “Up-to-seven” criteria and AFP of < 1000 ng/mL is more suitable in Chinese patients with HCC is unclear.

Most Chinese patients with HCC have a background of viral hepatitis and liver cirrhosis, which is significantly different from patients in Europe and the United States. Additionally, most Chinese HCC patients have significant differences in treatment options and their prognosis. Therefore, we conducted a retrospective study comparing the survival using “Up-to-seven” criteria to that using Milan and Hangzhou criteria, one of the most prevalent criteria in China, and compared the survival of patients that met and unmet the combination of “Up-to-seven” criteria and AFP of < 1000 ng/mL to explore an indication more suitable for Chinese patients. The results of this study may provide important references for doctors to select potential OLT candidates and improve survival in clinical work.

## Patients and Methods

This was a retrospective, single-center cohort study. Patients in this study all underwent liver transplantation and met the following inclusion criteria: (I) consecutive patients with HCC who underwent liver transplantation in a referral hospital from January 2015 to February 2019; (II) patients aged 18–70 years; (III) patients that were diagnosed with HCC before OLT. HCC diagnoses were based on liver pathology, imaging, and AFP levels, meeting Barcelona Clinic Liver Cancer (BCLC) diagnostic criteria (3). According to pathology, tumor size, tumor numbers, a sum of tumor diameter, and microvascular invasion were obtained. Exclusion criteria comprised (I) patients who underwent other organ transplantation, (II) patients who had other malignant tumors, (III) patients who did not meet the Hangzhou criteria, and (IV) patients with incomplete follow-up data. This study protocol conformed to the ethical guidelines of the 1975 Declaration of Helsinki and was approved by our hospital’s ethics committee.

According to the three OLT criteria, patients were divided into three groups. Group A met the Milan criteria, group B did not meet the Milan but met the “Up-to-seven” criteria, and group C did not meet the “Up-to-seven” but met the Hangzhou criteria.

According to the combination of “Up-to-seven” and AFP, patients were divided into two groups. Group 1 met the “Up-to-seven” criteria and AFP of < 1000 ng/mL, and group 2 did not meet “Up-to-seven” criteria or AFP of > 1000 ng/mL.

### Orthotopic Liver Transplantation (OLT) and OLT Criteria for HCC

For all patients, OLT was performed using the standard technique by excellent surgeons who have more than 20 years of OLT experience. All organs were from donation. The OLT criteria included Milan criteria, “Up-to-seven” criteria, and Hangzhou criteria.

Milan criteria included (I) single tumor diameter of ≤ 5 cm, (II) multiple tumor nodules of ≤ 3, and each nodule of ≤ 3 cm, and (III) no vascular invasion and metastases (4).

“Up-to-seven” criteria were the number of tumors plus the sum of tumor diameter of ≤ 7 and no vascular invasion (13).

Hangzhou criteria comprised (I) no major vascular invasion or extrahepatic metastasis, (II) total diameter of tumors of ≤ 8 cm or > 8 cm with preoperative AFP of ≤ 400 ng/mL, and histological studies with high or moderate differentiation (17).

### Follow-Up and Outcome

Up to 4 years after OLT, patients were regularly followed up, with an interval of 3–6 months. All patients received tacrolimus, mycophenolate mofetil, and prednisone after OLT. Prednisone was tapered and terminated one month after OLT. Tumor recurrence was referred to Barcelona Clinic Liver Cancer (BCLC) diagnostic criteria (3). The primary endpoint was OS, which was defined as the time from OLT to HCC-related death. HCC-related death was consequent of HCC recurrence, either intra- or extrahepatic, and led to a progressive worsening of performance status until death. The other causes of death were not defined as HCC-related death.

### Data Collection

The patients´ baseline demographic data were recorded, including sex, age, HCC etiology, tumor diameter, maximal tumor diameter, a sum of tumor diameters, preoperative AFP, vascular and microvascular invasion, HCC recurrence, survival time, and last follow-up status. A tumor diameter, maximal tumor diameter, and a sum of tumor diameters are the same in patients with one nodule. AFP stages were AFP stage 0 with AFP < 1000 ng/mL and AFP stage 1 with AFP > 1000 ng/mL. No patient with AFP = 1000ng/mL was in this study.

### Statistical Analysis

Continuous variables were reported as mean and standard deviation or median and interquartile range. Categorical variables were expressed as percentages. Besides the group allocation discussed above, patients were classified into two groups, death and non-death, and recurrence and non-recurrence. T-test was used to compare mean values, and Chi-squared test was used to compare categorical variables. Mann–Whitney test was used for comparison in the two groups, and Kruskal–Wallis test was used to compare the three groups. Univariate and multivariate analyses were performed based on the Cox proportional hazards regression model for the risk of OS (**Table 1**) and DFS (**Table 2**). Clinical features with a P of < 0.1 in the univariate analysis were included in the multivariate analysis. Kaplan-Meier curves and log-rank tests were used to assess differences in OS and DFS among the groups. Statistical analyses were performed using IBM SPSS 25 software. P of < 0.05 (two-sided) was considered statistically significant.

**Table 1:** Risk factors for HCC-related death on univariate and multivariate Cox regression analysis.

Variable	Univariate			Multivariate		
	HR	95%CI	*p*	HR	95%CI	*p*
Age	1.02	0.95–1.10	0.542			
Gender	1.43	0.30–6.74	0.650			
Etiology	0.71	0.35–1.47	0.358			
Pre LT RAF	0.33	0.04–2.55	0.285			
Pre LT TACE	1.36	0.40–4.67	0.625			
MELD	1.02	0.96–1.09	0.512			
BMI (kg/m^2^)	1.14	0.97–1.34	0.116			
AFP >1000 ng/ml	8.36	1.77–39.46	0.007	6.63	1.31–33.52	0.022
Tumor number	1.99	1.09–3.64	0.026	2.17	1.12–4.24	0.023
Max tumor diameter (cm)	1.32	1.12–1.56	0.001	1.33	1.08–1.62	0.006
Sum of tumor diameter (cm)	1.21	1.07–1.38	0.004	0.74	0.43–1.27	0.277
Microvascular invasion	3.67	0.77–17.42	0.102			
TNM Stage	2.03	1.20–3.41	0.008	1.44	0.66–3.15	0.362

LT, liver transplantation; RAF, radiofrequency ablation; TACE, transhepatic arterial chemotherapy and embolization; MELD, model for end-stage liver disease; BMI, body mass index; AFP, alpha-fetoprotein. Univariate and multivariate analyses were performed based on the Cox proportional hazards regression model. Clinical variables with a P of < 0.1 in the univariate analysis were included in the multivariate analysis.

**Table 2:** Risk factors for HCC recurrence on univariate and multivariate Cox regression analysis.

Variable	Univariate			Multivariate		
	HR	95%CI	*p*	HR	95%CI	*p*
Age	1.01	0.96–1.06	0.773			
Gender	0.80	0.18–3.47	0.797			
Etiology	0.90	0.56–1.44	0.660			
Pre LT RAF	1.12	0.40–3.12	0.827			
Pre LT TACE	1.67	0.63–4.42	0.307			
MELD	1.01	0.96–1.06	0.658			
BMI (kg/m^2^)	1.19	1.04–1.33	0.009	1.14	0.96–1.30	0.055
AFP >1000 ng/ml	6.66	1.93–23.04	0.003	10.05	2.45–41.13	0.001
Tumor number	2.10	1.31–3.23	0.002	2.08	1.25–3.47	0.005
Max tumor diameter (cm)	1.32	1.16–1.51	<0.001	1.25	1.07–1.46	0.005
Sum of tumor diameter (cm)	1.23	1.11–1.36	<0.001	0.85	0.60–1.20	0.366
Microvascular invasion	2.88	0.96–8.69	0.060	1.54	0.46–5.17	0.487
TNM Stage	1.88	1.27–2.78	0.002	1.16	0.61–2.18	0.652

LT, liver transplantation; RAF, radiofrequency ablation; TACE, transhepatic arterial chemotherapy and embolization; MELD, model for end-stage liver disease; BMI, body mass index; AFP, alpha-fetoprotein. Univariate and multivariate analyses were performed based on the Cox proportional hazards regression model. Clinical variables with a P of < 0.1 in the univariate analysis were included in the multivariate analysis.

## Results

### Patients’ Characteristics

All 153 patients were included in the present study. Among them, 136 (88.9%) were male, with a median age of 52.77 ± 0.65 years, and 17 (11.1%) were female, with a median age of 55.90 ± 2.76 years. Most of the HCC etiology was chronic hepatitis B (CHB) infection, which accounted for 71.2% (109/153) of cases. All patients were diagnosed with cirrhosis. The median maximal tumor diameter was 2.8 (1.5–4.5) cm. Among patients, 85.6% (n = 131) of them presented with < 2 nodules, and 14.4% (n = 22) of them presented with > 2 nodules. Of these patients with HCC, 57.5% (88/153) had microvascular invasion and a median AFP concentration of 8.0 (3.3–49.5) ng/mL. Patients who met the “Up-to-seven” criteria and AFP of < 1000 ng/mL were allocated to group 1. Group 2 comprised patients who did not meet the “Up-to-seven” criteria or those with AFP of > 1000 ng/mL, and they had larger tumor sizes and higher AFP levels than group 1 (**Table 3**). There were no differences in age, sex, model for end-stage liver disease (MELD) score, pre-liver transplantation (LT) radiofrequency ablation, or transhepatic arterial chemotherapy embolization between groups 1 and 2 (**Table 3**).

**Table 3:** Baseline of patients with HCC who met the “Up-to-seven” criteria and had AFP of < 1000 ng/mL.

Variables	Overall (n = 153)	Group 1* (n =121)	Group 2* (n = 32)	*P*
Demographic data				
Age (years)	53.0 (48.0, 58.0)	54.0 (48.0, 59.0)	53.0 (48.5, 56.0)	0.455
Sex Male Female	136 (88.9%) 17 (11.1%)	106 (87.6%) 15 (12.4%)	30 (93.8%) 2 (6.3%)	0.325
BMI (Kg/m^2^)	24.4 (22.7, 27.0)	24.3 (22.7, 26.7)	24.8 (22.8, 28.8)	0.143
Etiology of liver disease CHB CHC Alcoholic Others	109 (71.2%) 9 (5.9%) 24 (15.7%) 11 (7.2%)	85 (70.2%) 8 (6.6%) 18 (14.9%) 10 (8.3%)	24 (75.0%) 1 (3.1%) 6 (18.8%) 1 (3.1%)	0.620
MELD	11.0 (8.0, 14.0)	11.0 (8.0, 14.0)	12.5 (8.5, 17.0)	0.242
**Tumor characteristics**				
AFP values (ng/ml) < 1000 > 1000	146 (95.4%) 7 (4.6%)	121 (100.0%) 0 (0.0%)	25 (78.1%) 7 (21.9%)	<0.001
Tumor number ≦2 ≧3	131 (85.6%) 22 (14.4%)	111 (91.7%) 10 (8.3%)	20 (62.5%) 12 (37.5%)	<0.001
Max tumor diameter (cm)	2.8 (1.5, 4.5)	2.5 (1.5, 3.5)	6.0 (4.0, 8.5)	<0.001
Sum of tumor diameter (cm)	3.0 (1.6, 5.0)	2.5 (1.5, 3.6)	8.0 (4.9, 10.2)	<0.001
**Pre-LT treatment of HCC**				
Pre-LT RAF	34 (22.2%)	29 (24.0%)	5 (15.6%)	0.313
Pre-LT TACE	94 (61.4%)	74 (61.2%)	20 (62.5%)	0.890
**Explant pathology**				
Microvascular invasion	88 (57.5%)	60 (49.6%)	28 (87.5%)	<0.001
TNM stage I+ II III +IV	123 (80.4%) 30 (19.6%)	107 (88.4%) 14 (11.6%)	16 (50.0%) 16 (50.0%)	<0.001

*Group 1: met the “Up-to-seven” criteria and had AFP of < 1000 ng/mL. *Group 2: did not meet “Up-to-seven” criteria or had AFP of > 1000 ng/mL. BMI, Body mass index; CHB, chronic hepatitis B; CHC, chronic hepatitis C; LT, liver transplantation; RAF, radiofrequency ablation; TACE, transhepatic arterial chemotherapy embolization; MELD, model for end-stage liver disease; AFP, alpha-fetoprotein.

### Tumor-Related Characteristics May Be Closely Related to HCC Recurrence and HCC-Related Death

To explore factors associated with patient prognosis and risk factors for HCC recurrence, univariate and multivariate Cox regression analyses were performed on all patients. In the up to 4 years of follow-up, 20 (13.1%) patients had HCC recurrence. Univariate Cox regression showed that body mass index (BMI), AFP of > 1000 ng/mL, tumor numbers, maximal tumor diameter, a sum of tumor diameters, and TNM stage were the risk factors for HCC recurrence. Additionally, multivariate Cox regression suggested that tumor-related characteristics, such as AFP of > 1000 ng/mL, tumor numbers, and maximal tumor diameter, were the independent risk factors for recurrence (**Table 2**). Patients with recurrence were likely to have larger maximal tumor size, tumor count, and higher AFP concentration.

At the same time, we also performed univariate and multivariate Cox regression analyses on all patients for risk of HCC-related death. In the up to 4 years of follow-up, HCC-related death was reported in 11 (7.2%) patients. Univariate Cox regression showed that AFP of > 1000 ng/mL, tumor numbers, maximal tumor diameter, a sum of tumor diameters, and TNM stage were the risk factors for HCC-related death, and multivariate Cox regression revealed that tumor-related characteristics, such as AFP of > 1000 ng/mL, tumor numbers, and maximal tumor diameter were the independent risk factors for HCC-related death (**Table 1**).

### Tumor Recurrence and Mortality in Patients Who Met the “Up-to-Seven” Criteria Were Comparable to the Patients Who Met the Milan Criteria After OLT

**Figure 1:**
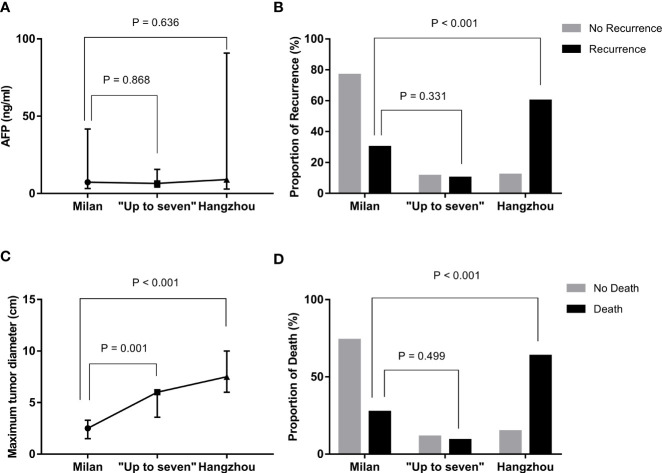
Differences in recurrence, death, and tumor characteristics in patients with HCC under distinctive liver transplant criteria. **(A)** compared with Milan criteria, patients who met “Up-to-seven” and Hangzhou criteria had similar AFP concentrations (*p* = 0.636). **(B)** compared with Milan criteria, patients who met “Up-to-seven” and Hangzhou criteria had a higher prevalence of HCC recurrence (*p* = 0.001). **(C)** compared with Milan criteria, patients who met “Up-to-seven” and Hangzhou criteria had longer tumor diameter (*p* < 0.001). **(D)** compared with Milan criteria, patients who met “Up-to-seven” and Hangzhou criteria had a higher prevalence of HCC-related death (*p* < 0.001). HCC, hepatocellular carcinoma; AFP, alpha-fetoprotein.

Based on the above-mentioned studies on tumor-related characteristics, we further analyzed the data of the three groups (groups A, B, and C) based on the three OLT criteria to explore whether OLT criteria are more suitable for Chinese patients (**Table 4**). By comparing the three groups, the results showed that the rates of HCC recurrence were 5.6% (6/108), 11.8% (2/17), and 42.9% (12/28), respectively, and the rates of HCC-related death were 2.8% (3/108), 5.9% (1/17), and 25.0% (7/28), respectively, in groups A, B, and C. Patients who did not meet the Milan but met the “Up-to-seven” criteria had longer maximal tumor diameter (P = 0.001) and a sum of tumor diameters (P < 0.001) than those who met the Milan criteria, but there was no significant difference in AFP concentration (P = 0.868), recurrence rates (P = 0.331), or HCC-related death rates (P = 0.499) (**Figure 1**). Patients who did not meet “Up-to-seven” but met the Hangzhou criteria had more advance tumor characteristics: larger tumor numbers (P < 0.001), longer maximal tumor diameter (P < 0.001), larger sum of tumor diameters (P < 0.001), and higher prevalence of microvascular invasion (P < 0.001), disease recurrence (P < 0.001), and HCC-related death (P < 0.001) than those who met the Milan criteria.

**Table 4:** Differences of patients with HCC under Milan, “Up-to seven,” and Hangzhou criteria.

Variables	MC^1^-in (n = 108)	MC-out and in-”Up-to-seven” (n =17)	“Up-to-seven”-out and in-HZ (n = 28)	*P*
**Demographic data**				
Age (years)	53.5 (48.0, 59.0)	54.0 (47.0, 57.0)	53.0 (48.5, 56.5)	0.721
Sex Male Female	93 (86.1%) 15 (13.9%)	16 (94.1%) 1 (5.9%)	27 (96.4%) 1 (3.6%)	0.232
BMI (kg/m^2^)	24.3 (22.6, 26.7)	24.8 (23.2, 26.9)	24.8 (22.8, 28.8)	0.311
Etiology of liver disease CHB CHC Alcoholic Others	75 (69.4%) 8 (7.4%) 17 (15.7%) 8 (7.4%)	13 (76.5%) 0 (0.0%) 2 (11.8%) 2 (11.8%)	21 (75.0%) 1 (3.6%) 5 (17.9%) 1 (3.6%)	0.654
MELD	11.0 (8.0, 14.0)	11.0 (9.0, 17.0)	14.0 (9.5, 17.5)	0.139
**Tumor characteristics**				
AFP values (ng/ml) < 1000 > 1000	104 (96.3%) 4 (3.7%)	17 (100.0%) 0 (0.0%)	25 (89.3%) 3 (10.7%)	0.181
Tumor number ≦2 ≧3	103 (95.4%) 5 (4.6%)	12 (70.6%) 5 (29.4%)	16 (57.1%) 12 (42.9%)	<0.001
Max tumor diameter (cm)	2.5 (1.5, 3.0)	4.0 (3.5, 6.0)	6.5 (5.5, 9.0)	<0.001
Sum of tumor diameter (cm)	2.5 (1.5, 3.5)	6.0 (5.0, 6.0)	8.5 (6.8, 10.7)	<0.001
**Pre-LT treatment of HCC**				
Pre-LT RAF	25 (23.1%)	4 (23.5%)	5 (17.9%)	0.821
Pre-LT TACE	66 (61.1%)	11 (64.7%)	17 (60.7%)	0.957
**Explant pathology**				
Microvascular Invasion	50 (46.3%)	12 (70.6%)	26 (92.9%)	<0.001
TNM Stage I+ II III +IV	99 (91.7%) 9 (8.3%)	12 (70.6%) 5 (29.4%)	12 (42.9%) 16 (57.1%)	<0.001

MC, Milan criteria; HZ, Hangzhou criteria; BMI, body mass index; CHB, chronic hepatitis B; CHC, chronic hepatitis C; MELD, model for end-stage liver disease; AFP, alpha-fetoprotein; LT, liver transplantation; RAF, radiofrequency ablation; TACE, transhepatic arterial chemotherapy and embolization.

### The “Up-to-Seven” Criteria Demonstrated Good Performance in OS and DFS of Chinese Cirrhotic Patients With HCC

**Figure 2:**
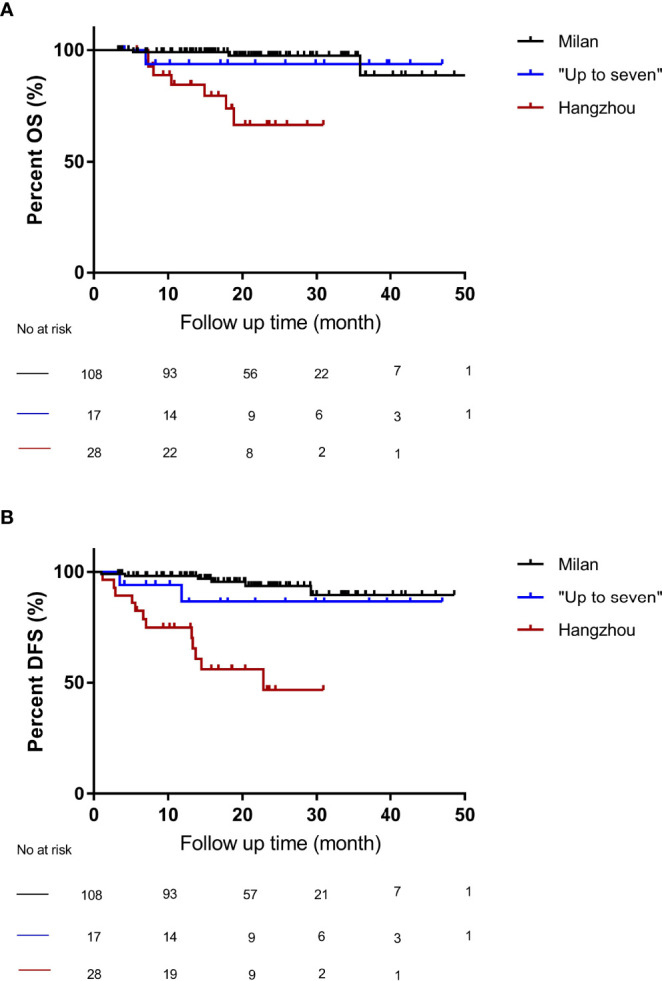
The OS and DFS of patients under different liver transplant criteria. **(A)** compared with the Milan criteria, patients who did not meet the Milan criteria but met the “Up-to-seven criteria” had no significant difference in OS (*p* = 0.69), while patients who did not meet the “Up-to-seven” but met the Hangzhou criteria had significant differences (*p* < 0.001). **(B)** compared with the Milan criteria, patients who did not meet the Milan criteria but met the “Up-to-seven criteria” had no significant difference in DFS (*p* = 0.35), while patients who did not meet the “Up-to-seven” but met the Hangzhou criteria had significant differences in DFS (*p* < 0.001). OS, Overall survival; DFS, disease-free survival; HCC, hepatocellular carcinoma.

To further demonstrate the performance of the “Up-to-seven” criteria, we performed a comparative analysis of OS and DFS among the three groups. The 1-, 3-, and 4-year OS of patients with HCC after OLT was 95.7%, 85.8%, and 85.8%, respectively, and DFS was 92.5%, 81.4%, and 81.4%, respectively, in groups A, B, and C. Further, we performed log-rank analyses on the OS and DFS among the three groups. Patients with HCC who did not meet the Milan but met the “Up-to-seven” criteria had no significant differences in OS (p = 0.69) (**Figure 2A**) and DFS (P = 0.35) (**Figure 2B**) from patients who met the Milan criteria, while the group who did not meet “Up-to-seven” but met the Hangzhou criteria was significantly inferior to the other two groups in terms of OS (P < 0.001) and DFS (P < 0.001) (**Figure 2**).

### The Combination of “Up-to-Seven” Criteria and AFP Showed Better Predictive Performance Than the Milan Criteria

To investigate the indications more suitable for Chinese patients, we combined the “Up-to-seven” criteria with AFP and investigated their significance in patient outcomes. Based on the above-described multivariate analyses, AFP was the independent risk factor for HCC recurrence and HCC-related death. Hameed B et al. reported that AFP of > 1000 ng/mL is a significant risk for HCC recurrence and suggested that patients with AFP of > 1000 ng/mL should be excluded from OLT in the Milan criteria (16). In 108 patients who met the Milan criteria and 125 patients who met “Up-to-seven” criteria in our study, AFP of > 1000 ng/mL was a significant risk for HCC recurrence (hazard ratio [HR]: 21.6; 95% confidence interval [CI]: 3.8–123.1; P = 0.001 and HR: 17.3; 95% CI: 3.4–88.6; P = 0.001, respectively). Therefore, we combined “Up-to-seven” criteria and AFP of < 1000 ng/mL to analyze the survival between patients who met and did not meet this criterium.

**Figure 3:**
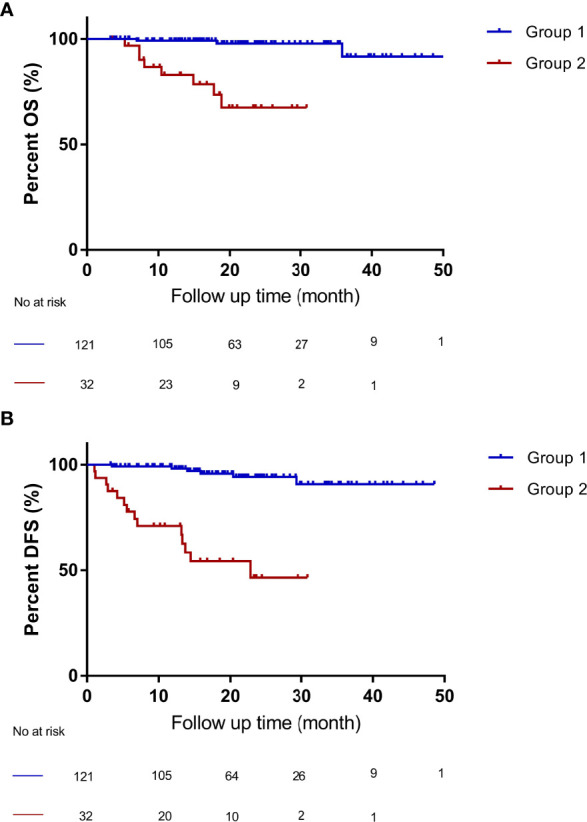
Compared OS and DFS of patients with HCC who met “Up-to-seven” criteria and AFP of < 1000 ng/mL (group 1) and patients who did not meet “Up-to-seven” criteria or had AFP of > 1000 ng/mL (group 2). **(A)** patients within group 1 had better OS (*p* < 0.001), with a 4-year OS of 91.6% than patients in group 2, with a 4-year OS of 67.5%. **(B)** patients within group 1 had better DFS (*p* < 0.001), with a 4-year DFS of 90.8%, than patients in group 2, with a 4-year DFS of 46.5%. OS, overall survival; DFS, disease-free survival, HCC, hepatocellular carcinoma. Group 1 (n = 121): patients who met the “Up-to-seven” criteria and had AFP of < 1000 ng/mL; group 2 (n = 32): patients who did not meet “Up-to-seven” or had AFP of > 1000 ng/mL.

According to the “Up-to-seven” criteria and AFP level, the patients were divided into two groups: patients who met the “Up-to-seven” criteria and had AFP of < 1000 ng/mL were classified into group 1 (n =121), and patients who did not meet the “Up-to-seven” criteria or had AFP of > 1000 ng/mL were in group 2 (n = 32). Then, Kaplan-Meier curves and log-rank tests were performed for the OS and DFS among groups 1 (n = 121) and 2 (n = 32). Patients with HCC who met the “Up-to-seven” criteria and had AFP of < 1000 ng/mL (group 1) had excellent survival with 4-year OS of 91.6% (P < 0.001) and DFS of 90.8% (P < 0.001), which was significantly better than group 2 (OS of 67.5%, DFS of 46.5%) (**Figure 3**) and patients who met the Milan criteria (OS of 89.8%, DFS of 89.6%).

The number of cases in group 1 was 121, which was larger than that in the Milan group (n = 108), which allowed 28.9% (13/45) of patients who did not meet the Milan criteria (n = 45) to still benefit from OLT and have better survival. The combination of “Up-to-seven” criteria and AFP differed significantly (HR: 18.9; 95% CI: 4.0–89.2; P < 0.001). Therefore, the combination of “Up-to-seven” criteria and AFP has good predictive performance, which is currently not reported in the literature.

## Discussion

The current study suggested that HCC patients who met the “Up-to-seven” criteria and AFP of < 1000 ng/ml had better survival than those who met the Milan criteria, allowing 28.9% (13/45) of patients who did not meet the Milan criteria to benefit from OLT.

Patients with HCC have high mortality, especially the high-risk individuals with cirrhosis and hepatitis B virus (HBV) infection. The optimal candidates for hepatic resection are patients with preserved hepatic function, Child-Pugh class A with normal bilirubin, and no portal hypertension. Otherwise, these patients have a high risk for HCC recurrence or liver failure (19–23). For the cases treated with systemic therapies, median survival has been only 1–1.5 years (19, 24–27). Patients with advanced cirrhosis, even presented with early-stage HCC, would have poor survival unless they had OLT. LT is the optimal option for unresectable patients with HCC who meet the Milan criteria. Milan criteria provide HCC patients with the same survival as the benign disease, with a 5-year survival of > 80%.

However, Milan criteria are restrictive, and only a few patients get an opportunity to be OLT candidates (5–7). More expanded OLT criteria have been explored to benefit more patients with HCC (17, 18, 28, 29). In 2001, Yao et al. proposed the UCSF criteria (14), which allowed approximately 20% of patients who did not meet the Milan criteria to benefit from LT. However, many studies have reported that patients with these expanded OLT criteria had lower OS and DFS than those who met the Milan criteria (7, 9). Given the severe organ shortages, the selection criteria are based on the utility principle assuring the maximal post-transplant survival rather than extended criteria that might lead to short-term survival. Especially in China, most patients diagnosed with HCC also have cirrhosis, and 70%–85% of them have been secondary to HBV infection (30). Hence, Chinese patients with HCC have a higher prevalence of HCC recurrence. Thus, looking for extended OLT criteria that have similar outcomes to the Milan criteria and could benefit more patients and reduce recurrence in China is vital.

The “Up-to-seven” criteria were proposed by Professor Mazzaferro, who also proposed the Milan criteria and the Metroticket 2.0 model. “Up-to-seven” criteria had excellent outcomes (13). Compared to Metroticket 2.0 model, “Up-to-seven” criteria are easy to calculate and do not need the internet, which is an advantage in developing countries. Now, it is used not only in the assessment of OLT but also in local and systemic treatment (31–34).

AFP is a critical biomarker for HCC recurrence and has been widely used in many noted models, such as the AFP model, the Hangzhou criteria, and the Metroticket 2.0 model. The combination of “Up-to-seven” criteria and AFP might be a good tool for selecting OLT candidates.

In this cohort study, 81.7% (125/153) of patients met the “Up-to-seven” criteria. In up to 4 years of follow-up, approximately 13.1% (20/153) of patients had HCC recurrence, and 7.2% (11/153) of patients had HCC-related death. “Up-to-seven” criteria had similar survival to Milan criteria and better outcomes than Hangzhou criteria. AFP is the independent risk factor for HCC recurrence and HCC-related death. The patients who met “Up-to-seven” criteria and had AFP of < 1000 ng/mL had excellent survival, with 4-year OS of 91.6% (P < 0.001) and DFS of 90.8% (P < 0.001), which is significantly better than in the other groups. This study suggests that Chinese cirrhotic patients with HCC who met the “Up-to-seven” criteria and had AFP of < 1000 ng/mL had excellent outcomes and should be offered the option of OLT.

This current study also compared the differences between the Milan, “Up-to-seven,” and Hangzhou criteria. Results showed that patients not meeting Milan but meeting “Up-to-seven” criteria had larger tumor size but a similar incidence of HCC recurrence and HCC-related death compared to Milan criteria, while those who did not meet “Up-to-seven” but met Hangzhou criteria had more advanced tumor characteristics and higher incidence of HCC recurrence and HCC-related death. That might partially contribute to the high proportion of microvascular invasion, representing 92.9% (26/28), in those who did not meet “Up-to-seven” but met the Hangzhou criteria group. The current study showed that Chinese cirrhotic patients with HCC who did not meet the Milan criteria but met “Up-to-seven” criteria also had good outcomes.

HCC recurrence and HCC-related death are important for long-term survival (35–37). Many risk factors exist for HCC recurrence and death after LT (38–44). In our center, patients with HCC recurrence had significantly higher AFP concentration, larger tumor size, vascular invasion, and poorer tumor pathological stage. The independent risk factors for HCC-related death after liver transplantation were AFP stage, tumor numbers, and maximal tumor diameter, which are consistent with the previous studies.

This study had certain limitations, including its retrospective nature, moderate case sample, and not performing the Metroticket 2.0 model. Milan criteria (45), “Up-to-seven” criteria, and the Metroticket 2.0 model all were proposed by Vincenzo Mazzaferro, and the Metroticket 2.0 model is good at predicting survival and calculated *via* the internet. However, this retrospective study aimed to look for expanded OLT criteria to benefit more patients. “Up-to-seven” criteria provide patients with good outcomes and are easy to calculate, and they do not need the internet, which makes it easy to use in clinical work, especially in developing countries. Furthermore, this study showed that “Up-to-seven” criteria and AFP of < 1000 ng/mL had remarkable outcomes.

In summary, Chinese patients with HCC usually had a high prevalence of cirrhosis and HBV infection, and those who met the “Up-to-seven” criteria and had AFP of < 1000 ng/mL had better OS and DFS than the other groups, including those who met the Milan criteria, allowing 28.9% of patients who did not meet the Milan criteria to benefit from OLT. Therefore, the combination of “Up-to-seven” criteria and AFP of < 1000 ng/mL might be the better strategy for LT in Chinese cirrhotic HCC patients. Additionally, this combination of criteria needs further validation *via* prospective study.

## Data Availability Statement

The raw data supporting the conclusions of this article will be made available by the authors, without undue reservation.

## Ethics Statement

The studies involving human participants were reviewed and approved by The Ethics Committee of the Fifth Medical Center of the Chinese People’s Liberation Army General Hospital. The ethics committee waived the requirement of written informed consent for participation.

## Author Contributions

Z-WL and H-BW performed the OLT. H-BW, X-DG, and Z-WL designed the clinical study and revised the manuscript, D-LZ and D-NF executed this study, analyzed data, and wrote the manuscript. XH and L-XL contributed to the statistical analysis. X-FN and Y-LZ contributed to data collection, analyzed data, and gave interpretation. All authors made significant contributions to this work. All authors read and approved the final manuscript.

## Conflict of Interest

The authors declare that the research was conducted in the absence of any commercial or financial relationships that could be construed as a potential conflict of interest.

## Publisher’s Note

All claims expressed in this article are solely those of the authors and do not necessarily represent those of their affiliated organizations, or those of the publisher, the editors and the reviewers. Any product that may be evaluated in this article, or claim that may be made by its manufacturer, is not guaranteed or endorsed by the publisher.
